# Avian hepatitis E virus infection of duck, goose, and rabbit in northwest China

**DOI:** 10.1038/s41426-018-0075-4

**Published:** 2018-05-02

**Authors:** Baoyuan Liu, Mengnan Fan, Beibei Zhang, Yiyang Chen, Yani Sun, Taofeng Du, Yuchen Nan, En-Min Zhou, Qin Zhao

**Affiliations:** Department of Preventive Veterinary Medicine, College of Veterinary Medicine, Northwest A&F University and Scientific Observing and Experimental Station of Veterinary Pharmacology and Diagnostic Technology, Ministry of Agriculture, China, Shaanxi 712100 Yangling, China

**To the Editor:** Hepatitis E virus (HEV) belongs to the Hepeviridae family, which consists of two genera, *Orthohepevirus* and *Piscihepevirus*^1^. Within the *Orthohepevirus* genus, four viral species, designated A–D, infect a wide range of mammalian species, including human, swine, wild boar, chicken, rat, ferret, rabbit, mongoose, camel, cow, and bat^1^. Within the species *Orthohepevirus* A, various HEV sequences are grouped into eight genotypes^2^. Genotypes 1 and 2 exclusively infect humans, whereas genotypes 3 and 4 infect humans and numerous other animal species^3^. Genotypes 5 and 6 have only been isolated from wild boar, whereas genotypes 7 and 8 have been isolated from camels^3^. Recently, it was recognized that strains of HEV genotypes 3 and 4 can cross-species barriers and are thus zoonotic genotypes^4^. In addition, it has been documented that HEV genotype 7 isolated from camels may also infect humans^5^. However, *Orthohepevirus* A genotypes 5, 6, and 8, as well as various HEV strains of *Orthohepevirus* C and D, have not yet been reported to cause zoonotic infections.

Avian HEV, a member of *Orthohepevirus* B, has been isolated from chickens with big liver and spleen disease, also known as hepatitis-splenomegaly syndrome^6, 7^. The avian HEV genome shares ~48% identity with mammalian HEVs^8^. In several previous studies, under experimental conditions, avian HEV could infect turkeys, but not rhesus monkeys or pigs^9^.

To investigate the prevalence of avian HEV infection in the northwest region of China, we collected sera, fecal swabs, and bile samples from a mixed group of animals consisting of 57 chickens, 30 ducks, 24 geese, and 16 rabbits cohabitating within a shared space in March 2017. Detection of antiviral serum IgG antibodies is often used as evidence of prior infection. We detected serum anti-avian HEV antibodies in four animal species by indirect ELISA using truncated avian HEV ORF2 protein as the coating antigen^10^ and a blocking ELISA protocol described by Liu et al.^11^ Based on the cutoff value of this method, the results showed that 20/57 chickens, 9/30 ducks, 6/24 geese, and 8/16 rabbits were positive for anti-avian HEV antibodies (Supplementary Figure S1). Because the results demonstrated the presence of specific anti-avian HEV antibodies in all species tested, avian HEV appears to infect chickens, ducks, geese, and rabbits within a mixed animal group.

As further confirmation of avian HEV infection of these four species, fecal swabs and bile samples were collected from the animals to test for avian HEV ORF1 and ORF2 RNA. First, total RNA was extracted from 100 μL of bile or from 10% suspensions of fecal swab samples using TRIzol Reagent (Invitrogen, Burlington, Ontario, Canada) followed by RT-nPCR. The two specific primer pairs used for RT-nPCR detection of avian HEV ORF1 and ORF2 RNA are described by Dong et al.^12^ and Sun et al.^13^, respectively. Fecal swab analysis detected 11/57 chickens, 8/30 ducks, 2/24 geese, and 2/16 rabbits positive for ORF1 (Supplementary Table S1). Additionally, we found 32/57 chickens, 12/30 ducks, 3/24 geese, and 6/16 rabbits positive for ORF2. From the bile samples, we identified 1/4 chickens, 1/4 ducks, 1/4 geese, and 1/4 rabbits positive for ORF1 (Supplementary Table S1). However, 2/4 chickens, 2/4 ducks, 1/4 geese, and 3/4 rabbits were positive using the ORF2 primers (Supplementary Table S1). Notably, the results showed that all positive samples for the *ORF1* gene were also positive for the *ORF2* gene. One possible reason for the increased detection of virus based on ORF2 in both fecal and bile samples is a higher mutation rate in ORF1 compared to ORF2 among the various avian HEV isolates^14^. PCR products of all positive samples were sequenced using an ABI 3730 Genetic Analyzer (JinSiTe Biotech Co., Nanjing, China) and analyzed using BLAST. The sequences (GenBank numbers MG922665, MG922666, MG922667 and MG922668 for *ORF1* genes, and MG922669, MG922670, MG922671 and MG922672 for *ORF2* genes) shared 97–100% identity with the various genomes of avian HEV strains in the GenBank database, and the phylogenetic analysis indicated that the viruses from the chickens, ducks, geese, and rabbits, designated CHN-SN-C2, CHN-SN-D2, CHN-SN-G2 and CHN-SN-R2, respectively, belonged to avian HEV strains (Fig. 1a). In addition, rabbit fecal swabs and bile samples all tested negative for HEV ORF2 RNA by RT-nPCR using the primer pairs described by Geng et al.^15^ Therefore, the RNA detection results indicate that avian HEV infected all species of animals in this mixed group, demonstrating cross-species transmission.

**Fig. 1:**
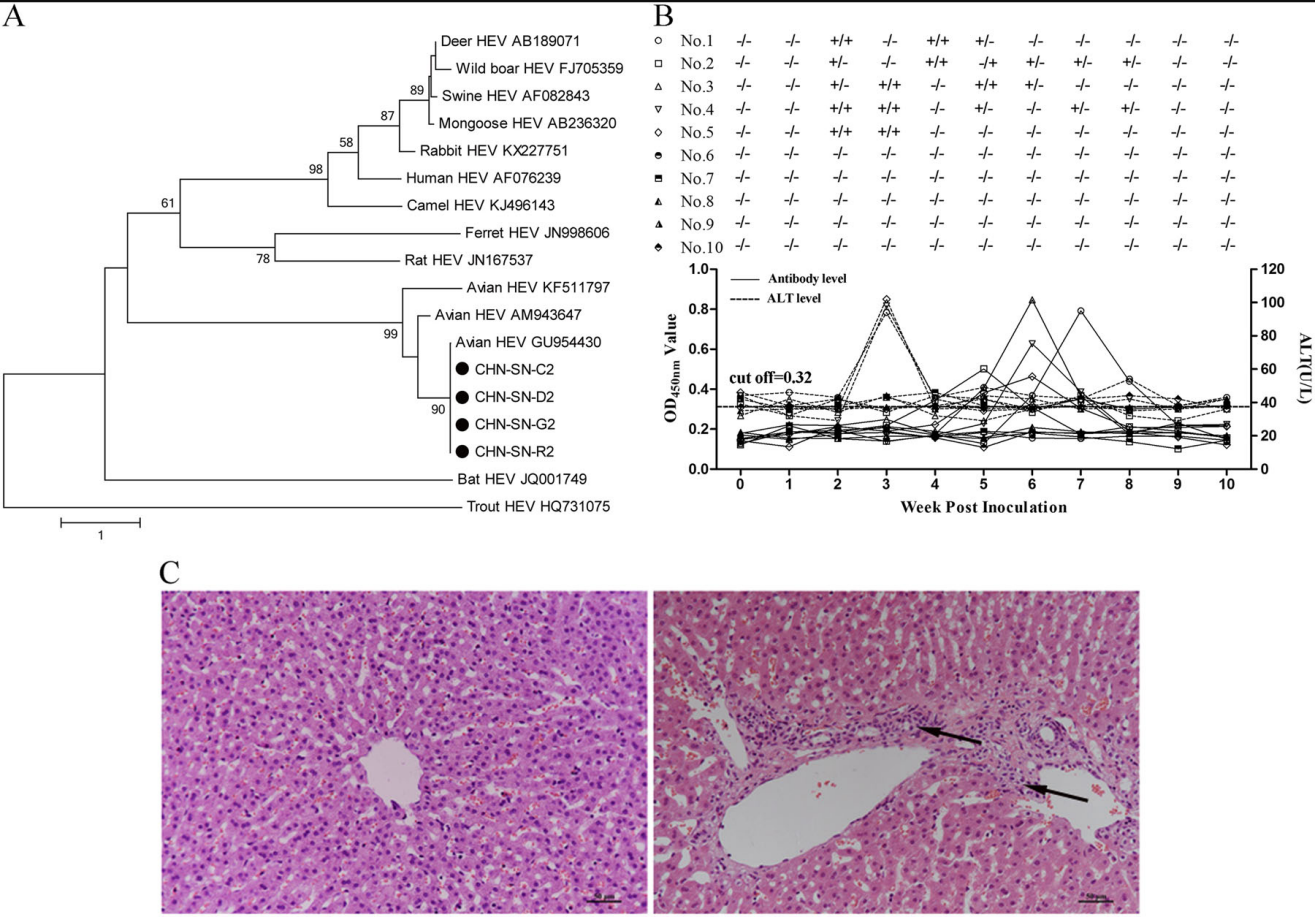
Avian HEV gene analysis and clinical evaluation of the virus infected rabbits **a** Phylogenetic trees based on the sequences of four partial of HEV *ORF2* genes (CHN-SN-C2, CHN-SN-D2, CHN-SN-G2, and CHN-SN-R2) isolated in this study and other HEVs from various animal species. The trees were generated by the neighbor-joining method with bootstrap tests of 1000 replicates using the MEGA 7.0 software. **b** Viremia/fecal viral shedding, ALT levels, and antibody levels in rabbits experimentally infected with CaHEV. **c** Microscopic lesions in livers from negative control rabbits (left) and rabbits experimentally infected with CaHEV (right) showed lymphocytic venous periphlebitis (arrows). Liver sections were stained with hematoxylin and eosin

To determine whether avian HEV infects rabbits, 10 6-week-old specific-pathogen-free New Zealand White rabbits were divided randomly into two groups (*n* = 5). Group 1 was inoculated intravenously with CaHEV (GenBank number GU954430) infectious stock (3 × 10^4^ GE per rabbit), and rabbits in group 2 were inoculated with PBS. Blood and fecal samples from each rabbit were collected prior to inoculation and weekly thereafter for 10 weeks post inoculation (wpi). Plasma samples from each rabbit were tested for alanine aminotransferase (ALT) levels, anti-avian HEV IgG antibodies, and avian HEV RNA using the methods described above. Fecal samples were also tested for avian HEV RNA. All rabbits were necropsied at 10 wpi, and liver samples were collected for pathologic evaluation. The results showed that all group 1 rabbits were positive for viremia or fecal virus shedding at 2 wpi and seroconverted between 4 and 8 wpi, and 3/5 rabbits demonstrated elevated ALT levels (94–102 U/L) at 3 wpi (Fig. 1b). None of the group 2 rabbits had viremia or fecal virus shedding, elevated ALT levels, or seroconversion throughout the study (Fig. 1b). Necropsies of 4/5 rabbits in group 1 demonstrated lymphocytic venous periphlebitis in the liver sections. In contrast, no visible pathological signs were observed in the livers from group 2 rabbits (Fig. 1c). Furthermore, we sequenced the avian HEV genomes recovered from the CaHEV-infected rabbits. The sequence data showed that the CaHEV recovered from the rabbits (designated CaHEV rabbit) and the CaHEV inoculum had the same genome structure and length and shared 98.9% nucleotide identity. CaHEV rabbit had 18 single mutation sites in the entire genome, which resulted in 12 nonsynonymous amino acid changes (Supplementary Table S2). Notably, ORF1 harbored 15 of 18 mutations, of which 10 mutation sites were mapped to the methyltransferase and RNA-dependent RNA polymerase domains within the ORF1 region (Supplementary Table S2).

In conclusion, avian HEV infection was demonstrated in a mixed animal group comprising chickens, ducks, geese, and rabbits in the northwest region of China by the detection of specific anti-avian HEV antibodies and avian HEV ORF1 and ORF2 RNA.

## Electronic supplementary material

Supplemental Table S1

Supplemental Table S2

Supplemental Figure S1

Distribution of percent inhibition (PI) of sera from chickens, ducks, geese, and rabbits in the mixed group using a blocking ELISA. The dotted lines represent the cut-off values of the blocking ELISA
