# Supplementary material for: Avian hepatitis E virus infection of duck, goose, and rabbit in northwest China
Source: Emerg Microbes Infect. 2018 May 2;7:76. doi: 10.1038/s41426-018-0075-4 (PMC5931602; doi:10.1038/s41426-018-0075-4)
Supplement: Supplementary file 1 — Supplemental Table S1 [file 41426_2018_75_MOESM1_ESM.docx]

**Supplementary Table S1** Detection of avian HEV RNA in fecal swabs and bile samples from chickens, ducks, geese, and rabbits from a mixed group of animals.

| Species | Fecal swabs | | Bile samples | | |  |
| --- | --- | --- | --- | --- | --- | --- |
|  | Sample No. | No. positive for avian HEV RNA ORF1/ORF2 | Sample No. | | No. positive for avian HEV RNA ORF1/ORF2 | |
| Chicken | 57 | 11/32 | 4 | 1/2 | |  |
| Duck | 30 | 8/12 | 4 | 1/2 | |  |
| Goose | 24 | 2/3 | 4 | 1/1 | |  |
| Rabbit | 16 | 2/6 | 4 | 1/3 | |  |
