# Supplementary material for: Avian hepatitis E virus infection of duck, goose, and rabbit in northwest China
Source: Emerg Microbes Infect. 2018 May 2;7:76. doi: 10.1038/s41426-018-0075-4 (PMC5931602; doi:10.1038/s41426-018-0075-4)
Supplement: Supplementary file 2 — Supplemental Table S2 [file 41426_2018_75_MOESM2_ESM.docx]

**Supplementary Table S2** Sequence analyses of complete genome of CaHEV recovered from rabbits.

| Nucleotide position | Genomic region | Nucleotide | | Amino acid | |
| --- | --- | --- | --- | --- | --- |
|  |  | CaHEV | CaHEV-Rabbit | Position | Substitution |
| 467 | ORF1-MeT | T | C | 151 | S-P |
| 655 | ORF1-MeT | A | G | 213 | Silent |
| 945 | ORF1-Y | C | T | 310 | S-F |
| 1013 | ORF1-Y | C | T | 333 | L-F |
| 1129 | ORF1-Y | C | T | 371 | Silent |
| 2051 | ORF1-X | G | A | 679 | G-S |
| 2152 | ORF1-X | C | T | 712 | Silent |
| 3393 | ORF1-RdRp | T | C | 1126 | V-A |
| 3566 | ORF1-RdRp | T | C | 1184 | S-P |
| 3588 | ORF1-RdRp | G | A | 1191 | G-E |
| 3907 | ORF1-RdRp | C | T | 1297 | Silent |
| 4064 | ORF1-RdRp | A | G | 1350 | T-A |
| 4310 | ORF1-RdRp | A | C | 1432 | M-L |
| 4316 | ORF1-RdRp | G | A | 1434 | V-I |
| 4586 | ORF1-RdRp | C | T | 1524 | P-S |
| 4653 | ORF3 | A | G | 2 | Y-C |
| 5751 | ORF2 | T | C | 350 | Silent |
| 5880 | ORF2 | T | C | 393 | Silent |
