# Supplementary figures and images for: Avian hepatitis E virus infection of duck, goose, and rabbit in northwest China
Source: Emerg Microbes Infect. 2018 May 2;7:76. doi: 10.1038/s41426-018-0075-4 (PMC5931602; doi:10.1038/s41426-018-0075-4)

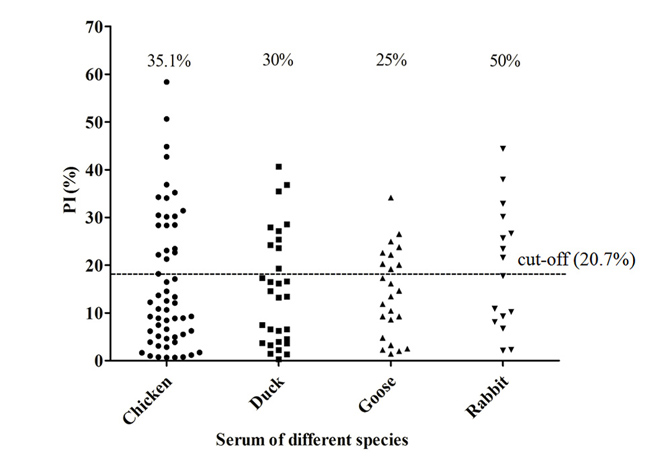

Supplement: Supplementary file 3 — Supplemental Figure S1 [file 41426_2018_75_MOESM3_ESM.jpg]
