# Supplementary material for: Avian hepatitis E virus infection of duck, goose, and rabbit in northwest China
Source: Emerg Microbes Infect. 2018 May 2;7:76. doi: 10.1038/s41426-018-0075-4 (PMC5931602; doi:10.1038/s41426-018-0075-4)
Supplement: Supplementary file 4 — Distribution of percent inhibition (PI) of sera from chickens, ducks, geese, and rabbits in the mixed group using a blocking ELISA. The dotted lines represent the cut-off values of the blocking ELISA [file 41426_2018_75_MOESM4_ESM.docx]

**Supplementary Figure S1.** Distribution of percent inhibition (PI) of sera from chickens, ducks, geese, and rabbits in the mixed group using a blocking ELISA. The dotted lines represent the cut-off values of the blocking ELISA.
